# Would shared decision-making be useful in breast cancer screening programmes? A qualitative study using focus group discussions to gather evidence from French women with different socioeconomic backgrounds

**DOI:** 10.1186/s12889-024-17876-5

**Published:** 2024-02-07

**Authors:** Laureline Guigon, Laura X. Gil Sánchez, Anne-Sophie Petit, Alice Le Bonniec, Partha Basu, Christelle M. Rodrigue, Marie Préau, Patricia Soler-Michel, Patricia Villain

**Affiliations:** 1 Early Detection, Prevention, and Infections (EPR) Branch, International Agency for Research on Cancer (IARC-WHO), 25 Avenue Tony Garnier, 69007 Lyon, France; 2 Unité Inserm, Université Lumière Lyon, 1296 « Radiations: Défense, Santé, Environnement », Lyon, France; 3 Centre Régional de Coordination des Dépistages des Cancers (CRCDC) Auvergne-Rhône-Alpes, Site Rhône & Métropole de Lyon, Lyon, France

**Keywords:** Breast cancer screening, Decision aid, France, Informed choice, Socioeconomic, Women, Shared decision-making, Focus group

## Abstract

**Background:**

To inform the development of an online tool to be potentially used in shared decision-making about breast cancer screening, French women were questioned about participation in breast cancer screening, the health professional’s role, and their perceptions of the proposed tool.

**Methods:**

We organised focus group discussions with 55 French women. Two different strategies were used to recruit women from high and low socioeconomic backgrounds. We applied both inductive and deductive approaches to conduct a thematic analysis of the discussions. We analysed the responses by using the main determinants from different health behaviour models and compared the two groups.

**Results:**

Independently of socioeconomic status, the most important determinant for a woman’s participation in breast cancer screening was the perceived severity of breast cancer and the perceived benefits of its early detection by screening. Cues to action reported by both groups were invitation letters; recommendations by health professionals, or group/community activities and public events were reported by women from high and low socioeconomic backgrounds, respectively. Among other positive determinants, women from high socioeconomic backgrounds reported making informed decisions and receiving peer support whereas women from low socioeconomic backgrounds reported community empowerment through group/community events. Fear of cancer was reported as a barrier in both groups. Among other barriers, language issues were reported only by women from low socioeconomic backgrounds; women from high socioeconomic backgrounds reported breast cancer screening-related risks other than overdiagnosis and/or overtreatment. Barriers to accessing the online tool to be developed were mainly reported by women from high socioeconomic backgrounds.

**Conclusion:**

Limitations in implementing shared decision-making for women from low socioeconomic backgrounds were highlighted. An online tool that is suitable for all women, regardless of socioeconomic status, would provide “on-demand” reliable and tailored information about breast cancer screening and improve access to health professionals and social exchanges.

**Supplementary Information:**

The online version contains supplementary material available at 10.1186/s12889-024-17876-5.

## Background

Breast cancer accounts for 11.7% (2.261 million new cases) of global cancer incidence and 6.9% (684,996 deaths) of global cancer mortality [1]. Breast cancer screening (BCS) has been proven to be effective in reducing breast cancer mortality. However, for this reduction in breast cancer mortality to be significantly effective, participation rates in BCS programmes should be at least 70%, which is still challenging for many countries [2, 3]. For example, in France where a programme has been implemented since 2004, the latest figures report a BCS participation rate of 47.7% in 2021–2022 [4]. A general drop in BCS uptake has been observed for all age groups and all regions in France since 2012 [5]. This was due to controversy about the benefits and harms of BCS and the risk of overdiagnosis and overtreatment. This issue was reported globally by the media, but the negative impact was particularly felt in France [6, 7]. To address the negative publicity, an action plan to revamp France’s BCS programme was put in place in 2017 by the French Ministry of Health and the French National Cancer Institute (INCa) [8]; the plan was informed through broad public and scientific consultation [9–11]. The plan's priority was to ensure that women were able to make an informed decision/choice about their participation in BCS. Therefore, a greater involvement from health professionals was deemed to be necessary. As defined in the literature, for a choice or decision to be “informed”, it must be consistent with an individual’s values and based on adequate information including risks [12]. Shared decision-making (SDM) was identified by INCa as a promising strategy to support women to make an informed decision about BCS, and which could be integrated into consultations [13]. SDM has been described as an interactive and balanced steps-based discussion between health professional and patient, usually using a decision aids (DAs), in order to help patients in making a decision regarding a health issue [14, 15]. DAs are evidence-based tools explicating the decision to be taken and empowering patients by making the choice more personal, clearer and easier to understand [16–18]. They have been shown to be effective to support informed choice in decision-making about cancer screening including BCS [18–21]. SDM has been reported to be difficult to implement in busy medical practices. Some authors suggest that informed choices should be made prior to medical appointments, reserving SDM to those who need it the most [22–25]. Developing an online DA tool that supplements SDM is therefore an interesting proposal [26]. This idea is supported by a recent review showing that web-based DAs were effective to increase informed choice about BCS [27]. To inform the development of such a tool a patient-centred approach is strongly recommended. This is based on the guidelines of the International Patient Decision Aid Standards (IPDAS) group, i.e. the authoritative body regulating DAs [16, 28, 29]. It is also recommended when developing any implementation strategy to increase the reachability of the target population [30–32]. In French settings, this approach was deemed to be essential. Indeed, there was a need to update the evidence on the psychosocial determinants of BCS participation, which was scarce and was essentially gathered more than seven years ago in France [10, 11, 33, 34]. Psychosocial determinants have been reported to be barriers of and/or facilitators to women’s participation in BCS in France or other settings. For example, among those determinants, fears of breast cancer diagnosis and/or mammography, perceptions and beliefs about breast cancer, risk perception, lack of knowledge, negative experiences, or lack of motivation/awareness were reported as main barriers [11, 33, 35, 36], whereas mammography habits, fear of breast cancer, motivation, social support, self-efficacy, or positive intention were reported as examples of facilitators [33, 35, 37, 38]. In France, evidence was also lacking regarding the impact of women’s socioeconomic status (SES) on the psychosocial determinants of BCS. SES indicators are captured at the individual or area-based level. At the individual level, SES indicators most commonly include measures of income, education, and occupational social class [39]. Participation in BCS, in France and other settings, has indeed been shown to be linked to a woman’s SES, with the most disadvantaged women being less likely to participate [40–42]. More precisely, for example, a French study has reported that women with low incomes or having lived in a precarious situation were less likely to participate in BCS than women from higher socioeconomic backgrounds [43]. The use of a conceptual framework investigating psychosocial determinants of BCS can provide a more comprehensive overview. Different key theories or models have been applied to investigate determinants of health behaviour including BCS. Those are for example, the health belief model [44], the empowerment theory [45–47], social support [48], and the theory of interpersonal behaviour [49]. Such models are of great interest given that their key variables or constructs encapsulate the majority of the psychosocial determinants of BCS identified in the scientific literature [33, 35–38]. Moreover, the health belief model has been widely used to study those determinants [37, 44]. Here we report the results of our study. Our main objectives were (i) to explore the psychosocial determinants of French women’s participation in BCS including the role of health professionals, and (ii) to investigate to what extent a woman’s socioeconomic background influences these determinants. As secondary objective, our study aimed to inform on the utility of an online tool to be used in SDM and/or as DA in the BCS programme in France. This was achieved by (i) gathering the women’s general perceptions about one potential tool presenting those characteristics and (ii) discussing our overall findings in the perspective of developing such a tool.

## Methods

We followed the Standards for Reporting Qualitative Research (SRQR) guidelines (see Supplementary Table 1, Supplementary Material 1) [50].

### Participants

To participate in the study, women had to comply with the following main eligibility criteria: (i) residing in France, (ii) aged 45-74 years, and (iii) with no prior history of breast cancer. In total, 10 focus group discussions (FGDs) were conducted, and women were recruited in two ways. Primary recruitment was through the Seintinelles digital platform, which aims to facilitate collaboration between researchers and individuals in breast cancer research studies (https://www.seintinelles.com/). The FGDs, which were initially planned as face-to-face meetings, were conducted online in April 2020, due to the lockdown for the Covid-19 pandemic. The FGD guide was adapted for online sessions and the recruitment, planned originally to be limited to the Auvergne-Rhône-Alpes (AuRA) region, was extended to the whole of France. The French eligible women who were able to join online, were invited to participate in our study. Out of 76 volunteers, 40 participants, i.e. seven FGDs, were set up; for each FGD, we tried to achieve a balance between women who had had no or few mammograms, and women who attended regular screening.

Our study was originally planned to be conducted only with Seintinelles participants. However, because our results showed that the Seintinelles women had high levels of education and employment, we decided to extend our study to women from low socioeconomic (LSE) background. In a second recruitment phase, women from LSE backgrounds were recruited with the support of two collaborators of the Centre Régional de Coordination des Dépistages des Cancers (CRCDC) from the AuRA region. As for other CRCDCs in other French regions, the CRCDC AuRA organises and implements the BCS programme in the AuRA region. Those CRCDC AuRA’s collaborators, i.e. “Atelier santé ville”/Saint-Fons and Atelec/“Centre social Le Lavoir”, work with vulnerable women/populations from Saint-Fons and Ambérieu-en-Bugey respectively. These two towns are in the AuRA region and are located in geographical areas identified with prominent low SES populations [51]. Our collaborators recruited French-speaking women among their participants, through face-to-face invitations. Because the women were recruited through associations working with vulnerable populations and from two LSE geographic areas, they were expected to be most likely from LSE backgrounds. The invited women met the main eligibility criteria. Due to recruitment issues, only 15 women were recruited and participated in three face-to-face FGDs; one was conducted in Saint-Fons (in June 2020) and two were conducted in Ambérieu-en-Bugey (in 2021).

### Focus group discussions (FGDs)

All women, prior to participating in the FGDs, provided both written and verbal consent to participate in the study. The FGD guide (see Supplementary Material 1) and the methodology were piloted with French IARC colleagues (not scientists), aged 50–55 years.

FGDs involving five or six participants and lasting an average duration of 120–150 minutes, were led by a trained moderator (LG or PV) in French. Extra time was added to LSE FGDs to repeat and re-formulate questions when women had challenges with the French language. Questions raised by the women, or misconceptions based on misinformation (which were raised among the Seintinelles women only), were addressed at the end of the session by PV, so that participants left the discussions with clear, complete and correct information. Anonymous questionnaires about education level and profession were also completed by the participants to collect information on SES (see Supplementary Material 1). All FGDs were video-recorded and professionally transcribed verbatim, except for the two FGDs conducted in Ambérieu-en-Bugey; these transcriptions were led directly by two researchers (LGS and PV) because the women’s speech was difficult to understand. After anonymisation, a thematic analysis was undertaken by making a continuous comparison of the data and codes using NVivo (QSR international software (v. 11)). Both inductive and deductive approaches were used to identify determinants of BCS participation (detailed below). To increase the validity of the results, double coding was performed; for the Seintinelles FGDs, coding was performed by one researcher (LG) with double coding by ALB and PV (five FGDs in total); for LSE FGDs, coding was performed by two different researchers (LG, LGS) with double coding by PV (all FGDs). Consultations and consensus meetings took place to review and refine the main codes used; by LG, ALB and PV (FGDs with HSE women) or by LG, LSG and PV (FGD with LSE women).

### FGDs analysis

We first analysed the FGDs from the Seintinelles women. We applied both inductive and deductive approaches to conduct the thematic analysis, which was performed in two steps. First, we used an inductive method and generated codes and then themes directly from the data. Second, through internal discussions, we arranged the identified themes into broader groups by using a minimum set of determinants/constructs. The choice of these determinants was guided both by the themes themselves and by the scientific literature; they were based on the main theories/models and/or concepts used to explain or understand health behaviour [52]. Besides generic determinants usually found in theories/models such as “Perceived barriers” and “Facilitators”, we identified more specific determinants from different theoretical and/or conceptual frameworks. Those determinants are: “Perceived susceptibility, severity and benefits” and “Cue to action” from the health belief model [44], “Individual empowerment” and “Community empowerment (female social support)” from the empowerment theory [45–47] and social support [48], and “Habits and past experiences” from the theory of interpersonal behaviour [49] and consistent with a literature review [53] (Table 1). Thematic analysis of the FGDs from the LSE women was performed in two steps. We first performed inductive analysis to define codes and themes. Then, we used the conceptual framework that emerged from the Seintinelles FGDs analysis, to report themes and determinants (Table 1).

In “Community empowerment (female social support)” in Table 1, we reported themes related to empowerment through female social support. We differentiated themes referring to “Peers (women)” from themes referring to “Group/community activities”.

The “Peers (women)” category was described as support from female social networks; that was resulting in individual positive actions to inform and/or encourage, as well as sharing experiences and testimonials. “Group/community activities” were referring to activities in which several women were engaged to be informed about BCS. In France, those activities are mainly conducted by the CRCDCs and collaborators and target women from LSE backgrounds to tackle inequalities in access to cancer screening. For examples, “Atelier santé ville”/Saint-Fons and Atelec/“Centre social Le Lavoir” organise group activities with vulnerable women/populations from Saint-Fons and Ambérieu-en-Bugey respectively.

The frequency of a theme was reported in regard to the number of FGDs (n) in which the theme was reported and discussed (Table 1). The themes identified through Seintinelles FGDs analysis were arranged in descending order of frequency, with the most frequent themes reported first (Table 1). The table was then completed with the themes from the LSE group and compared against the Seintinelles results. In the LSE group, the themes related to “Perceived susceptibility, severity and benefits” and “Cue to action” were more frequently reported.

### SES indicators

To capture the socioeconomic backgrounds of the women participating in FGDs, women’s education level and occupational social class were used as indicators [39]. Women were categorised as high socioeconomic background (HSE) when they had a high education level (i.e. with baccalaureate and university years) and an intermediate to high employment position. Women were categorised as low socioeconomic background (LSE) when they had no school diploma or a school diploma below “baccalaureate” and were either unemployed or with no intermediate to high employment position [39]. Based on this definition, the Seintinelles women were categorised as HSE women; women from Ambérieu-en-Bugey and Saint-Fons were categorised as LSE women.

## Results

A total of 40 Seintinelles participants took part in our study. They were from different locations in France (not shown) including urban and rural settings (see Supplementary Material 1). The women were aged 54.5 years on average and 62.5% (*n* = 25) had a family history of breast cancer. They generally had a high socioeconomic background; 67.5% (*n* = 27) had a high education level (i.e. with three university years or more) and 70% (*n* = 28) were employed. Within the employed subgroup, 71.4% (*n* = 20) had an intermediate to high employment position (see Supplementary Material 1). All those women, except one, reported participating in BCS and/or being in favour of BCS (not shown). Barriers (“Perceived barriers”, e.g. fear of pain or cancer diagnosis, fear of false results (Mistrust in results), radiation risks (Risk related to radiations) and issues with access to health professionals (Getting an appointment…)) were reported, but less frequently than the positive determinants (Table 1). Among the reasons given to participate in BCS, the most frequently reported was that BCS is the main way to decrease or eliminate the risk of developing breast cancer, i.e. by treating it early if necessary (Screen early to treat early). The reason generally associated was gaining “peace of mind”. Breast cancer was indeed perceived as a tangible concern essentially due to a family history of breast cancer (Table 1). Awareness of the potential signs of breast cancer (Potential symptoms), recommendations by health professionals, having a family history of breast cancer and receiving an invitation letter were reported as factors encouraging participation in BCS (Cue to action) (Table 1). Health professionals, mainly general practitioners and gynaecologists were recognised as experts that women trust (see Supplementary Material 1). Women generally expect their health professionals to know their personal health history well and to provide detailed information about BCS; this empowers them to make informed decisions (Informed choice) (Table 1 and Supplementary Material 1). Being accustomed to health procedures, notably gynaecological procedures and/or having had a mammography (Habits and past experiences: Health habits/health practices, Experience with mammography), as well as positive experiences related to BCS (Habits and past experiences: Positive past experience with mammography) were also identified as factors that would facilitate/encourage women to participate in BCS. Among those factors, individual and community empowerment were also highlighted (Table 1). Individual empowerment was reported through the duty of taking care of one’s health (To be responsible for one’s one health) initiating discussions with health professionals (To take the lead) and making an “informed choice”. Community empowerment was from peers (Peers (women), i.e. through female social support networks (Table 1); that was resulting in individual positive actions to inform and/or encourage, as well as sharing experiences and testimonials (Examples/testimony). When a potential online tool to be used in a SDM process and/or as a DA was presented to the women, they reported that this would be interesting. Indeed, it would be a good way to facilitate access to health professionals (see Supplementary Material 1). However, the reported barriers were (i) the Internet and social media themselves, e.g. may provide misinformation, (ii) differential use of social networks and Internet due to age, and (iii) absence of needs as they did not feel socially isolated.

A total of 15 women from Ambérieu-en-Bugey and Saint-Fons were recruited with help from the CRCDC AuRA. They were 52 years old on average and only one woman quoted a family history of breast cancer (see Supplementary Material 1). Considering that the women were recruited through associations working with vulnerable populations and from two LSE geographical areas (Methods), they were expected to be most likely from LSE backgrounds. This was confirmed by the analysis of their education level and employment status. Indeed, all women reported either no diploma (*n* = 4), below the “baccalaureate” (i.e. equivalent to a high school diploma or Alevels) or other diploma (*n* = 2) or preferred not to report (*n* = 9); 66.7% (*n* = 10) reported being unemployed, and four being retired. None of the participants reported intermediate to high job status.

All women in our LSE group reported being in favour of BCS with the majority reporting participating in BCS (not shown). Barriers to BCS (Perceived barriers) were less frequently reported than positive determinants; they were mainly related to fear of receiving a cancer diagnosis and French language issues (Table 1). Pain was mentioned but was not reported as a barrier to BCS participation. Among the positive determinants of BCS, the perceived benefits of BCS as “peace of mind”, and receiving “cues to action” were more frequently reported (Table 1). Both susceptibility to breast cancer, and severity of breast cancer were reported but mainly related to cancer in general; some women reported a feeling of fatalism (“one day one could catch it (cancer)”, one FGD) and/or examples of close family members with cancer (one FGD). As “cues to action”, a BCS invitation letter and group/community activities organised by CRCDC AuRA or other bodies, and/or television advertisements, were more frequently reported. Two characteristics of the letter were frequently highlighted: receiving it at home and/or regularly at a specific time of the year. In one FGD only, recommendations from health professionals were mentioned as “cues to action”. Instead, the health professional’s role was described as mainly providing information and helping to overcome barriers,essentially fear: “must not force her…but give her courage” (FGD2R), health professionals need to “convince but not force” (FGD1P1). Two women reported that health professionals helped them make their first appointment for mammography (see Supplementary Material 1). Other positive determinants of BCS were community empowerment through “group/community activities”. Through those activities, women reported receiving information about BCS and encouragement to participate. Individual empowerment was reported mainly through “to take the lead” (Table 1). This was, for example, to make an appointment or to seek information if required to understand the invitation letter and to get information about BCS. A potential online tool to be used in a SDM process and/or as a DA was reported to be interesting by the LSE women. Indeed, they reported that it would facilitate meeting other women, improve access to health professionals, and help to get information if needed: “for someone who has doubts, who knows nothing (regarding BCS)" (FGD3P2). Barriers related to general Internet/social media use were reported in one FGD (see Supplementary Material 1). Favouring the implementation of online DA, use of WhatsApp groups/Facebook *via* mobile phones with Arabic-speaking women was highlighted in another FGD (FGD3).

**Table 1:** Women’s main psychosocial determinants associated with breast cancer screening

Determinants†	Main themes		Seintinelles’ women (N^§^=7)		LSE women (N^§^=3)	
			n^§^	Quotations^¶^	n^§^	Quotations^¶^
**Perceived susceptibility, severity and benefits**	Family history of breast cancer		7	“I had a mother who had breast cancer at a young age…, I said to myself… I need to be monitored very regularly” (FGD526)		NA
	Screen early to treat early		7	"For mee, it is a warranty to treat early enough"(FGD715)		NA
	Peace of mind		5	“It ensures peace of mind to do it [screening]. Otherwise, you are in uncertainty that perhaps you are developing cancer and you don’t know it” (FGD102)	3	“The important thing is to control” (FGD2B)
**Cue to action**	Recommendations from health professionals*		6	“I am followed by a gynaecologist… who told me… it is time to start mammograms” (FGD116)	1	“[My] gynecologist told me to do everything,… do every exam, do a mammogram, colorectal [cancer screening test]” ​​(FGD3M
	Potential symptoms		6	“Because while feeling the breasts, I felt a lump” (FGD127)	1	“It makes me burn…pain I went to see the doctor" (FGD2R)
	Family history of breast cancer		3	“I have been getting screened for two, three years already, for having a history of cancer in the family” (FGD419)		NA
	Invitation letter		3	“Afterwards, I admit that if there had not been the letter, I would not have done it” (FGD307)	3	“It’s good, it comes home, it’s important…to control…it’s better” (FGD3R) “It’s the date, it’s the date every year…it comes to everyone.” (FGD2R)
	Group/community or TV adverts			NA	3	“We had a meeting in the room here… one day she showed me the letter that I received in the mailbox…that helps” (FGD2R) “[At]TV [adverts]when they advertise to go, to see” (FG1P6)
**Facilitators**	Free of cost		5	“I have gone because it was free” (FGD559)	2	“Well, it’s free. So it’s true that it encourages doing so” (FGDP3)
**Habits ** **and past experiences**	Health habits/health practices: heath care, health professionals, screening other than BCS		6	“It’s one of those things like smears that ultimately… we end up getting used to” (FGD217)		NA
	Positive past experience with mammography		5	“I have had several mammograms, well, the exam is not pleasant, but I cannot say that it is painful” (FG214)		NA
	Experience with mammography		4	“I am used to get screened since the age of 50” (FGD556)	1	“The gynecologist made me an appointment for a mammogram, it was for the first time, now I’m doing everything [by myself]” (FGD2N) “Afterwards we get used to it” (FGD2Be)
**Individual empowerment**	To be responsible for one’s own health		6	“It’s also up to everyone to take responsibility for their own health” (FG446)	1	“You have to go get help and not stay in your corner” (FGD2O)
	To take the lead and initiate discussion with health professionals or other related		6	“It was I who informed my doctor of the cases that were in the family” (FGD633)	2	“Finally, it was me who made an appointment for a check-up. But really only for that” (FG1P4 “If she is not capable [of understanding] you have to ask…now there is everything, there is the association…if you ask nothing…she has to look for a solution” (FG2R)
	Informed choice		5	“I think it’s good that everyone can actually make their decision and be informed of what exists” (FG715)		NA
**Community** **empowerment** ** **(female social support)**	Peers (women) **	Inform	6	“I think it’s the role of every woman to inform a friend, a colleague, or people” (FGD313)		NA
		Examples / Testimony push	5	“She [her colleague in remission] tells me, if you don’t want to go through that, if you don’t want to experience what I experienced, well do it ” (FGD343)		
		Encourage	3	“We will perhaps find more arguments to convince someone to have a mammo[graphy] than a professional” (FGD367)		
	Group/ community activities**	Inform		NA	3	“To discuss about breast cancer screening: “at Atelec *** yes…at home no” (FGD3P3)
**Perceived barriers**	Related to health professionals*	Getting an appointment, and/or enough time for discussion	5	“It’s a hassle to get doctors. We are lacking doctors” (FG367)	1	“Sometimes general practitioners don’t take the time to discuss this ” (FGD1P4)
	Fear	Pain	4	“[The mammogram] still hurts relatively… we’re not going to do that lightly”(FG343)		NA
		Cancer diagnosis	3	“What would prevent me from getting tested is the fear…of the result” (FGD217)	3	“It’s fear” [of the result] (FGD1P2)
	French language issues			NA	2	“The problem is we don’t understand well, we don’t speak well, we don’t write well” (FGD3R)
	Risk related to radiations		3	“We receive rays at the time we do this examination.… it’s not necessarily a good thing” (FGD5fl)		NA
	Mistrust in results		3	“Because we know that there are false negatives, false positives” (FGD631)		NA

NA: not applicable; LSE: low socioeconomic background; BCS: breast cancer screening; FGD: focus group discussion.

Women were recruited either through the Seintinelles digital platform (“Seintinelles”) or with the help of the CRCDC AuRA ( “LSE”). The themes identified through Seintinelles FGDs analysis were arranged in descending order of frequency, with the most frequent themes reported first. The frequency of a theme was reported in regard to the number of FGDs (n) in which the theme was reported and discussed. In the LSE group, the themes related to “Perceived susceptibility, severity and benefits” and “cue to action” were more frequently reported.

CRCDC: Centre Régional de Coordination des Dépistages des Cancers; AuRA: the Auvergne Rhône-Alpes region of France.

†All positive except barriers; barriers were defined as determinants that could hold back women from participating in BCS. Determinants were from the main health models/theories/concepts, e.g. the health belief model [44], the empowerment theory [45–47], social support [48] and the theory of interpersonal behaviour [49].

^§^ N: the total number of FGDs conducted; n: the total number of FGDs in which the identified theme was discussed. For Seintinelles women, quotations were reported only when *n* ≥ 3; for LSE women, due to the lower number of conducted FGDs, we also reported the theme when *n* = 1 but reported by at least two women.

^¶^One example of quotation representative of each theme was reported in general; the complete sentence and other examples are reported in supplementary tables (Supplementary Material 1).

* General practitioners or gynaecologists mainly.

** In “Community empowerment (female social support)”, we reported themes related to empowerment through female social support. We differentiated themes referring to “Peers (women)” from themes referring to “Group/community activities”. The “Peers (women)” category was described as support from female social networks; that was resulting in individual positive actions to inform and/or encourage, as well as sharing experiences and testimonials. “Group/community activities” were referring to activities in which several women were engaged to be informed about BCS. In France, those activities are mainly conducted by the CRCDCs and collaborators and target women from LSE to tackle inequalities in access to cancer screening.

*** Atelec: French association aiming to teach French to the immigrant population; the CRCDC AuRA is used to organise activities with them to talk about organised cancer screening.

## Discussion

Almost all our study participants (independently of SES) had a positive attitude towards BCS and reported participating and/or being in favour of BCS. This was also reflected through the limited number of barriers that they reported compared with the positive determinants of BCS. It is possible that recruitment through the Seintinelles digital platform or CRCDC AuRA might have positively altered women’s perceptions of BCS. For example, the Seintinelles digital platform aims to facilitate collaboration between scientists studying BCS and the public; it is therefore very possible that a woman’s interest in this website is driven by her family history of breast cancer. This was indeed reported in the HSE group to explain breast cancer susceptibility and as a “cue to action”. Our results could have been different with women who were against or more hesitant towards BCS, even though recruitment of such women to participate in our study would have taken subsequent additional time. This was notably due to the Covid context and difficulties in recruiting LSE women to participate in our study. Therefore, caution should be exercised to generalise our results to all HSE women and all LSE women, including those against or hesitant towards BCS. We invite the reader to keep this in mind, especially in the following section, where we will discuss the results in detail. However, one point should be highlighted in regard to the key psychosocial determinants emphasised here, including differences and similarities between socioeconomic groups.Those could reflect a “thinking process” that led women to develop positive attitudes towards BCS (e.g. thinking that breast cancer or cancer in general is a threat might enhance the perceived benefits of screening). In this sense, our results could be very much informative for public health officers to develop new initiatives to improve BCS.

We report and put into perspective the psychosocial determinants of BCS from the point of view of two groups of women from different socioeconomic backgrounds. That was enabled by using a conceptual framework to extract and analyse the determinants. This conceptual framework encapsulates our study’s findings. They show that the key psychosocial determinants associated with women's participation in BCS were, independently of the SES, (i) perceived susceptibility, severity and benefits i.e. perceptions of cancer or breast cancer as a threat and BCS as beneficial to treat early or for peace of mind, (ii) cue to action, (iii) individual empowerment, and (iv) community empowerment. Those reflect the psychosocial determinants reported in France and other settings [11, 33–38]. However, our results provide a comprehensive overview, even though this could be specific to the French setting and/or to our specific groups of women. Putting into perspective the psychosocial determinants of BCS from the point of view of two groups of women, from different socioeconomic backgrounds, highlighted interesting similarities and differences. Cues to action reported by both groups were invitation letters; recommendations by health professionals, or group/community activities and public events were reported by women from high and low socioeconomic backgrounds, respectively. Women’s empowerment seemed to be differently reported in our study. HSE women reported making informed decisions (individual empowerment) and receiving peer support through female social networks (Community empowerment). LSE women did not report making informed decisions (Individual empowerment) and reported community empowerment through group/community events. Group activities are mainly conducted by the CRCDCs to target women from LSE backgrounds to tackle inequalities in access to cancer screening in France. Our results reinforce the importance of those interventions to provide information and knowledge to LSE women [54, 55].

Our results also highlight the importance of the BCS invitation letter regardless of the woman’s socioeconomic background. Particular emphasis on the importance of the letter was reported by women from LSE backgrounds. It is most likely that this a consequence of group/communities activities conducted by the CRCDC, again reinforcing the importance of such activities. Our results also stressed the necessity of translating the invitation letter and diffusing translated information related to BCS. Indeed, the French language was reported as a barrier for women in our LSE group. This language barrier may be specific to our group of LSE women and related to a possible recruitment bias. However, a similar result was reported following a broad public consultation about BCS in 2016 in France [9]; diffusion of the information materials into languages other than French to increase reachability of women was highlighted [9, 54]. Providing translated information might contribute to tackle inequalities in access to BCS. As in other settings, participation in BCS in France has indeed been shown to be linked to a woman’s SES, with the most disadvantaged women being less likely to participate [40–43]. This is particularly relevant because the poverty rate has been shown to be higher in France in the immigrant population than in the non-immigrant population. In France, in 2022, the immigrant population was 10.3% of the total population; in 2019 the poverty rate was 31.5% among the immigrant population versus 12.8% among the non-immigrant population [56].

Our study was originally designed to document the development of an online tool to be used in SDM. Our results provide new insights into the implementation of such tools, and DA tools in general, in the BCS programme in France. That was enabled though a more detailed analysis of our overall results, through the lens of our findings related to “empowerment”. Empowerment is a muti-level construct defined as giving people more control over the decisions and actions that impact their lives [45, 46]. In our FGDs, both individual empowerment (here more psychological) and community empowerment were identified [45–47]; both were reported in our study as positive determinants of BCS participation. However, community empowerment through “peers”, i.e. individual women as a source of information, knowledge, and encouragement in BCS, was reported only in the HSE group and not in the LSE group. Community empowerment though “Group/community activities” was reported only in the LSE group. These results again strengthen the necessity to continue group/community activities organised by the CRCDCs and their collaborators to inform LSE women about BCS [54, 55]. Individual/psychological empowerment was reported in the LSE group; however, “informed choice/decision” was only reported among the women from HSE backgrounds. These findings are novel considering the 2016 national BCS consultation, when the impact of a woman’s socioeconomic background was not studied [9–11]. Our results suggest that the concept of informed choice/decision may not be salient in our LSE group and/or this might not be crucial in their BCS decision-making process compared with women in our HSE group. Our findings are consistent with the literature that an individual’s empowerment and decision-making process is different according to SES; people from LSE backgrounds “shifting one’s focus towards meeting immediate needs and threats” [45, 57, 58]. It is interesting to highlight that although this was not formally assessed, women from LSE backgrounds who participated in our study seemed to have reached, as did the women from HSE backgrounds, an “informed choice/decision” about BCS. They showed a positive attitude which seemed to be based on adequate knowledge and the majority reported participation in BCS [12]. In the absence of available DAs for France’s BCS programme, these results suggest that (i) both an invitation letter and community/groups activities are effective to inform LSE women, and (ii) women from LSE backgrounds might not need as much or such complex information (as provided in traditional DAs) to make informed choices about BCS [16–18, 59]. This finding is supported by another study highlighting that informed choice in screening via DAs may be difficult to achieve due to the complexity of the information presented [60]. Some levels of BCS information should not even be provided if a woman does not specifically ask for it, because this may cause unnecessary stress and worry. For example, in our study women from LSE backgrounds did not report potential risks related to BCS; only one woman from a HSE background mentioned risks related to overdiagnosis and/or overtreatment. This latest result might suggest that risks related to BCS overdiagnosis and/or overtreatment may no longer be salient among women as they were during the controversy in France [7], even though a French study performed two years prior to our study paints a different picture [61]. The term “informed choice”, when referring to participation in cancer screening programmes may itself generate anxiety causing the public to question whether health professionals themselves have doubts about the BCS programme [60, 62].

Evidence reported here and in other settings has shown that SDM, notably in cancer prevention, might be difficult to implement due to time constraints in medical practice [22–25, 61, 63]. Our results add that it might also be particularly challenging to implement for women from LSE backgrounds. In addition, the health professional’s role, which is key in SDM, seems to be perceived differently depending on the woman’s socioeconomic background. In our study, only women from HSE backgrounds reported the health professional’s recommendation as a cue to action and key to empowerment through informed decision-making as described previously [9, 38]. Besides issues with the French language, this could be explained by women from LSE backgrounds having a different relationship with their health professionals due to their SES [64] and - as we found - less preventive health practices. Participation in BCS has been shown to be closely linked to women’ health practices, which are shaped by women’s cultural and societal background [37, 65, 66].

Our study highlights some conflicting results. DA and SDM could be implemented among women from HSE backgrounds, but would have limited impact among women from LSE backgrounds in the current health system. Implementation of DA and SDM should therefore be carefully considered to avoid further amplifying the existing inequalities already affecting BCS participation [40–43], although one study did show that SDM tended to benefit the disadvantaged women more than the advantaged women [67].

Suboptimal BCS participation rates suggest that there is room for improvement in delivering information about BCS [4, 5]. Besides translating information into different languages, our results support the development of an online tool [26]; in our study, women showed interest in this idea, even though barriers, mainly in the HSE group, were highlighted.

To address our study’s results and the aforementioned issues, we suggest that this online tool have the following specific features. First and foremost, the information provided through this tool should be on-demand [68]. Women should be free to decide whether they want information on BCS. They should also be able to “tailor” the information themselves, when needed, filtering the type and amount of information needed. This can be achieved by providing different levels of information, with basic/minimum information being provided at level zero as previously suggested [60]. Second, the online tool should facilitate access if needed to health professionals to discuss BCS. Finally, our results suggest that the tool should also facilitate communication with peers; this was reported by women among our LSE group [26]. We are finalising a systematic review that supports this proposal. The online nature of the tool renders the implementation of the required features feasible [69]. When developing and implementing this tool, we strongly believe that users from LSE backgrounds will not be disadvantaged. On the contrary, we strongly believe that the development of such a tool should be seen as a possible means to explore/highlight specific cognitive skills that women living in LSE conditions have been forced to acquire [58].

Our study presents three limitations that might be interesting to address in future studies. Firstly and mainly, caution should be exercised to generalise our results to all HSE and all LSE women, i.e. including those women who are against or more hesitant towards BCS. Secondly, it was not possible to know which responses were from women who participated in the organised national BCS programme or in the opportunistic BCS [70, 71]. Finally, empowerment, e-health or health literacy were not formally assessed using validated scales; SES, even though assessed trough education, employment, and place of living, was not assessed based on income.

The strengths of this study are its conformity with recommended qualitative study guidelines, strong methodology, sufficient sample size, and the two recruitment strategies enabling us to report on the role of SES in women's perceptions towards BCS.

## Conclusion

Our study explored the psychosocial determinants of BCS, notably by focusing on the impact of socioeconomic background on those determinants. This was performed from a different approach than women’s health literacy or e-health literacy. Our results highlight the limitations in using SDM and DA notably for women from LSE backgrounds; the importance of continuing group/community activities and providing translated information to tackle inequalities in access to cancer screening were also highlighted. Developing an on-demand online tool that women can access to obtain reliable and accurate information that is tailored to their needs, improving peer support and access to health professionals, are all essential to reach as many women as possible. Our results will be of benefit not only to France’s national BCS programme but also to other BCS programmes globally.

### Electronic supplementary material

Below is the link to the electronic supplementary material.

Supplementary Material 1

## Data Availability

Supplementary material (Supplementary Material 1.pdf) is provided; the document contains additional information about both the methods used and the women who participated in our study including additional quotations. The complete qualitative datasets generated and/or analysed during the current study are not publicly available, because the data contain information that could compromise the research participants' privacy/consent but are available from the corresponding author upon reasonable request.

## Abbreviations

AuRA: Auvergne-Rhône-Alpes
BCS: Breast cancer screening
CRCDC: Centre Régional de Coordination des Dépistages des Cancers
DAs: Decision aids
FGDs: focus group discussions
HSE: High socioeconomic
INCa: Institut National du Cancer, France
IPDAS: International Patient Decision Aid Standards
LSE: Low socioeconomic
SES: Socioeconomic status
SDM: Shared decision-making
SRQR: Standards for Reporting Qualitative Research
