# Supplementary material for: Would shared decision-making be useful in breast cancer screening programmes? A qualitative study using focus group discussions to gather evidence from French women with different socioeconomic backgrounds
Source: BMC Public Health. 2024 Feb 7;24:404. doi: 10.1186/s12889-024-17876-5 (PMC10851553; doi:10.1186/s12889-024-17876-5)
Supplement: Supplementary file 1 — Supplementary Material 1 [file 12889_2024_17876_MOESM1_ESM.pdf]

Supplementary Material

1. Additional information about methods.. 2

1.1. Standards for Reporting Qualitative Research (SRQR) guidelines. 2

1.2. Focus group discussion grid and methodology 4

2. Additional information i.e., characteristics and quotations in French, related to the Seintinelles' women.. 5

2.1. Participants' characteristics.. 5

2.2. Additional quotations regarding breast cancer screening 6

2.3. Participants' and expectations of health professionals' (mainly general practitioners and gynecologist) role in breast cancer screening 9

2.4. Perception of the online tool... 10

3. Additional information i.e., characteristics and quotations in French, related to women from low socioeconomic background.. 12

3.1. Participants' characteristics... 12

3.2. Additional quotations 13

1. Additional information about methods

1.1. Standards for Reporting Qualitative Research (SRQR) guidelines.

Supplementary Table 1

No.	Topic	Item	Page† or section
**S1**	Title	Concise description of the nature and topic of the study identifying the study as qualitative or indicating the approach (e.g., ethnography, grounded theory) or data collection methods (e.g., interview, focus group) is recommended	**Page 1**
**S2**	Abstract	Summary of key elements of the study using the abstract format of the intended publication; typically includes background, purpose, methods, results, and conclusions	**Page 1**
**S3**	Problem formulation	Description and significance of the problem/phenomenon studied; review of relevant theory and empirical work; problem statement	**Introduction section**
**S4**	Purpose of research question	Purpose of the study and specific objectives or questions	**Introduction section**
**S5**	Qualitative approach and research paradigm	Qualitative approach (e.g., ethnography, grounded theory, case study, phenomenology, narrative research) and guiding theory if appropriate; identifying the research paradigm (e.g., postpositivist, constructivist/ interpretivist) is also recommended; rationale**	**Method section**
**S6**	Researcher characteristics and reflexivity	Researchers' characteristics that may influence the research, including personal attributes, qualifications/experience, relationship with participants, assumptions, and/or presuppositions; potential or actual interaction between researchers' characteristics and the research questions, approach, methods, results, and/or transferability	**NA**
**S7**	Context	Setting/site and salient contextual factors; rationale**	**Introduction and Method section**
**S8**	Sampling strategy	How and why research participants, documents, or events were selected; criteria for deciding when no further sampling was necessary (e.g., sampling saturation); rationale**	**Method section**
**S9**	Ethical issues pertaining to human subjects	Documentation of approval by an appropriate ethics review board and participant consent, or explanation for lack thereof; other confidentiality and data security issues	**Ethics approval statement**
**S10**	Data collection methods	Types of data collected; details of data collection procedures including (as appropriate) start and stop dates of data collection and analysis, iterative process, triangulation of sources/methods, and modification of procedures in response to evolving study findings; rationale**	**Method section**

**S11**	Data collection instruments and technologies	Description of instruments (e.g., interview guides, questionnaires) and devices (e.g., audio recorders) used for data collection; if/how the instrument(s) changed over the course of the study	**Method section section and additional information**
**S12**	Units of study	Number and relevant characteristics of participants, documents, or events included in the study; level of participation (could be reported in results)	**Results section and additional information**
**S13**	Data processing	Methods for processing data prior to and during analysis, including transcription, data entry, data management and security, verification of data integrity, data coding, and anonymization/de-identification of excerpts	**Method section**
**S14**	Data analysis	Process by which inferences, themes, etc., were identified and developed, including the researchers involved in data analysis; usually references a specific paradigm or approach; rationale**	**Method section and author contributions sections**
**S15**	Techniques to enhance trustworthiness	Techniques to enhance trustworthiness and credibility of data analysis (e.g., member checking, audit trail, triangulation); rationale**	**Method section**
**S16**	Synthesis and interpretation	Main findings (e.g., interpretations, inferences, and themes); might include development of a theory or model, or integration with prior research or theory	**Results section**
**S17**	Links to empirical data	Evidence (e.g., quotes, field notes, text excerpts, photographs) to substantiate analytic findings	**Results section**
**S18**	Integration with prior work, implications, transferability, and contribution(s) to the field	Short summary of main findings; explanation of how findings and conclusions connect to, support, elaborate on, or challenge conclusions of earlier scholarship; discussion of scope of application/generalizability; identification of unique contribution(s) to scholarship in a discipline or field	**Discussion section**
**S19**	Limitations	Limitations - Trustworthiness and limitations of findings	**End of discussion section**
**S20**	Conflicts of interest	Potential sources of influence or perceived influence on study conduct and conclusions; how these were managed	**Conflicts of interest section**
**S21**	Funding	Sources of funding and other support; role of funders in data collection, interpretation, and reporting	**Funding section**
*The authors created the SRQR by searching the literature to identify guidelines, reporting standards, and critical appraisal criteria for qualitative research; reviewing the reference lists of retrieved sources; and contacting experts to gain feedback. The SRQR aims to improve the transparency of all aspects of qualitative research by providing clear standards for reporting qualitative research.			
**The rationale should briefly discuss the justification for choosing that theory, approach, method, or technique rather than other options available, the assumptions and limitations implicit in those choices, and how those choices influence study conclusions and transferability. As appropriate, the rationale for several items might be discussed together.			

† For other pages than the first one, we cited "section" instead of page number to avoid numbering mistakes.

1.2. Focus group discussion grid and methodology

The FGD session was divided into three main parts to cover the following items related to breast cancer screening (BCS): the invitation letter, the role of health professionals, and women's perceptions regarding the online SDM/DA potential tool "CANELLES".
To initiate and facilitate discussions, participants were presented with specific material related to each part to mimic a play role: the invitation letter sent by the CDRC AuRa, cards presenting different possible roles of health professionals (mainly general practitioners) , flyers mimic how to reach the site through invitation letter and the tool by itself. Those were digitized for the online sessions with Seintinelles' women.

Supplementary Table 2

**Stage/theme**	**Support activity**	**Duration**
Receptions of participants, connexion (video and audio) to the session, reminder of information on ethical consent, and presentation of each member and the course of the session		15 min
Invitation letter	screening invitation letter	20 min
Relationship with the health professional	Cards Classification activity and discussion of the role of health professionals in screening decision making	15 min
Online shared decision support tool	Presentation of a sample tool and discussion on the use of this online tool	30 min
Closing discussions, summarizing, and filling out the questionnaire		5 min
Answering questions, correcting misinformation, and thanking the participants		10 min

2. Additional information i.e., characteristics and quotations in French, related to the Seintinelles' women

2.1. Participants' characteristics

Supplementary Table 3

		Number of women	%
**Total number**		40	
**Average age**	54,5 (8,1)		
**Place of residency**			
Dans un village		10	25
Dans une ville		19	47.5
Dans une grande ville		10	25
Sans réponse		1	2.5
**Familial status**			
Mariée/ en couple/ pascsée		28	70
Célibataire		4	10
Divorcée/séparée		7	17.5
Veuve		1	2.5
**With children**			
Oui		30	75
Non		10	25
**Education/School diploma**			
BEP/CAP		2	5
BAC		3	7.5
BAC+2/DUT/BTS/BTSA		8	20
BAC+3		5	12.5
BAC+4		7	17.5
BAC+5		15	37.5
**Occupation**			
Cadre ou profession intellectuelle supérieure		10	25
Profession intermédiaire		6	15
Profession libérale		4	10
Employée		8	20
Retraitée		7	17.5

Sans emploi	5	12.5
**With a general practitioner**		
Oui	40	100
Moyenne du nombre de consultations par an 3,1		
**Gynaecological follow-up**		
Oui	37	92.5
Non	3	7,5
**Family risk of breast cancer**		
Oui	25	62.5
Non	14	35
Ne sait pas	1	2.5

2.2. Additional quotations regarding breast cancer screening

Supplementary Table 4

N	Determinants	Main themes	Quotations
1	**Perceived susceptibility, severity and benefits**	Screen early to treat early	« Pour moi c'est une garantie de traiter suffisamment tôt. » FGD715<br>« (..) être en mesure de soigner plus vite si quelque chose était détecté » FGD446
		Family history	« Moi, je disais ça parce qu'en fait, moi, j'ai eu une mère qui a eu un cancer du sein jeune,… je me suis dit oh bah tiens, faut que je me fasse surveiller très régulièrement » FGD526<br>"(…) je suis à risque, c'est-à-dire que moi, je me fais dépister depuis deux, trois ans déjà, pour avoir des antécédents de cancers du sein dans le famille (…)" FGD419
		Peace of mind	« Pour moi, c'était important de le faire (…) Ça assure une tranquillité de le faire (le dépistage). Sinon vous êtes dans l'incertitude que peut-être vous êtes en train de développer un cancer et que vous le savez pas » FGD102<br>"Alors moi, j'ai été amenée à en faire (des mammographies) (…) et du coup, c'est pour s'assurer que tout va bien" FGD211
2	**Cue to action**	Recommendations from health professionals	«je suis suivie par (une) gynéco (…) qui m'a dit à un moment, ben voilà, maintenant, il est temps de

			commencer les mammographies, et c'était avant de recevoir le courrier (lettre d'invitation) » FGD116
		Potential symptoms	« (…) personnellement, j'ai déjà eu mammographie, échographie, parce que en palpant les seins, j'ai senti une boule (…) » FGD127
		Family history of breast cancer	« (…) j'ai des antécédentes familiaux dans mon entourage proche qui fait que moi je fais des mammo depuis l'âge de trente-cinq ans » FGD 142<br>« (...) je me fais dépister depuis deux, trois ans déjà, pour avoir des antécédents de cancers dans la famille, aussi bien chez ma maman que chez mes tantes » FGD419<br>« (…) j'ai des antécédentes familiaux dans mon entourage proche qui fait que moi je fais des mammo depuis l'âge de trente-cinq ans » FGD 142<br>« (...) je me fais dépister depuis deux, trois ans déjà, pour avoir des antécédents de cancers dans la famille, aussi bien chez ma maman que chez mes tantes » FGD419<br>« (…) j'ai été amenée à en faire parce que ma maman a eu un cancer du sein y a cinq ans » FGD211<br>"(...) j'en fais régulièrement tous les deux ans, compte tenu que dans ma famille, ma mère a été touchée par le cancer du sein (...)" FGD214
		Invitation letter	« Après, moi, j'avoue que si y avait pas eu la lettre, moi, je l'aurais pas fait (…) » FGD307
3	**Facilitators**	Free of cost	« J'y suis allée aussi parce que c'était gratuit » FGD559
4	**Habits and past experiences**	Health habits/health practices: heath care, health professionals, screening other than BCS	« Pour moi, ça fait partie des choses comme les frottis auxquels finalement, quand on va chez le gynécologue, on finit par s'habituer » FGD217
			« Est-ce que ça voudrait dire que nous qui sommes déjà familiarisées avec ce qui est un dépistage (...) lorsqu'on reçoit ce courrier, on le comprend vite » FGD440
		Positive past experience with mammography	« (…) Moi, j'ai eu plusieurs mammographies, bon, l'examen, n'est pas agréable, mais je peux pas dire que ce soit douloureux » FGD214
		Experience with Mammography	« Moi (…) depuis l'âge de trente-cinq ans (…) à cette époque, c'était tous les ans, une mammographie, et puis après (…) le dépistage automatique depuis l'âge de cinquante ans » FGD556
5	**Individual mpowerment**	To be responsible for one's own health	« Et puis bon, chacun doit un petit peu se responsabiliser aussi hein » FGD127<br><br>« C'est aussi à chacun de se responsabiliser pour sa propre santé » FGD446

		To take the lead and initiate discussion with health professionals		« Moi, j'en ai parlé parce que c'est moi qui ai prévenu mon médecin des cas qu'il y avait dans la famille. Et j'ai demandé ce qui pouvait être fait en termes de dépistage » FGD633
		Informed choice		« Je pense que c'est bien que chacun puisse effectivement prendre sa décision et soit informé de ce qui existe » FGD715
**6**	**Community empowerment (female social support)**	Peers (women)	Inform	« (…) je pense qu' c'est un peu le rôle de chaque femm' d'informer une amie, une collègue, ou des personnes » FGD313
			Examples / Testimony push	« (...) par rapport à ma collègue qui est en rémission (…) elle me dit, si vous voulez pas passer par là, si vous voulez pas vivre ce que moi j'ai vécu, bah faites-le (…) » FGD343
			Encourage	« On trouvera peut-être plus d'arguments pour convaincre quelqu'un de faire une mammo qu'un professionnel » FGD367<br>« Je pense que c'est plus le rôle de gens effectivement, autour de soi (…) et qui parlent de leur propre expérience, est c'est plus convainquant que le professionnel » FGD330
**7**	**Perceived barriers**	Fear	Pain	«(mammography) fait quand-même relativement mal… c'est pas de gaîté de coeur qu'on va faire ça » FGD343
			Cancer diagnosis	« (Ce qui m'empêcherait de me faire dépister c'est) la peur (…) du résultat » FGD217<br><br>« (…) je pense que la peur du diagnostic peut être un gros frein » FGD1Del
		Risk related to radiations		« Y a des femmes aussi qui pensent que d'avoir trop de radio peut être nocif aussi » (FGD453)<br><br>« (…) on reçoit des rayons au moment où on fait cet examen. Donc on accumule les rayons dans son corps et c'est pas forcément une bonne chose » FGD5fl
		Mistrust in results		« (…) Parce qu'on sait qu'il y a des faux négatifs, des faux-positifs (…) Donc chaque fois que j'y vais, je me demande ce que je ferais si on me dit, ah bah oui, y a quelque chose » FGD631

BCS : breast cancer screening

2.3. Participants' and expectations of health professionals' (mainly general practitioners and gynecologist) role in breast cancer screening

Supplementary Table 5

	**Main themes (n)†**	**Quotations**
**Perceptions**	Limited access (n=5)	« C'est une galère pour avoir des médecins. On est en manque » FGD367 « Enfin, moi, mon médecin généraliste m'a jamais dit, est-ce que vous avez fait des mammographies ? » FGD102
	Experts they trust (n=5)	« Pour moi, il représente l'autorité. Il a autorité, pas dans le sens dictatorial (…) C'est lui qui sait. (…) une autorité de compétence » FGD603 « Moi, je me suis mise dans les mains de mon médecin. (...) je fais confiance totalement en ce qu'elle me dit de faire » FGD116
	Experience, age and gender (n=3)	« Moi, ce que je recherche, c'est surtout l'expérience (…) le médecin qui a vu au cours de sa carrière de multiples cas, ben, moi, j'ai plus confiance » FGD307 « Moi, j'avoue, que j'ai un médecin qui est très compétent (…) Déjà, c'est une femme, donc y a moins de barrières (…) » FGD313
**Expectations**	Guide an informed decision/choice (n=5)	« C'est-à-dire qu'à un moment-donné, c'est quand-même à eux de nous dire, voilà, je vous explique et ensuite, c'est votre décision, bien entendu, mais je serai toujours là à vos côtés y a pas de soucis » FGD405 « Son rôle (…) c'est de donner l'information, une information neutre, qui permet à la femme de prendre sa décision, qui lui est propre » FGD419
	Explain/inform about the screening process (n=5)	« (...) y a des gens qui peut-être parce qu'ils ont peur (…) enfin, pour toutes sortes de raisons, auront besoin d'une vraie discussion (...) et pour moi, cette question-là (…) est dans le fait, d'expliquer les arguments, de détailler l'examen pour rassurer sa patiente » FGD217
	Personalized support and care (n=3)	« (...) c'est quand-même un peu le B, A, Ba, je dirais de connaître un peu son patient et de pouvoir l'aider éventuellement si y a des antécédents, ou même pour information » FGD367
	Recommend screening (n=3)	« Mais la gynécologue, si vous y allez, effectivement, il est de son rôle entre quarante et cinquante ans de le proposer, bien évidemment » FGD214

† (n): number of focus groups out of the total number (N=7) in which the theme was mentioned.

2.4. Perception of the online tool

Supplementary Table 6

Perceptions	Main themes (n)†	Quotations
**Barriers / Negative aspects**	Spreading of misinformation (n=7)	« N'importe qui peut mettre n'importe quoi n'importe où. Et c'est pas au sein d'une structure médicale (…) » (FGD307)<br>« Sur internet il y a tout et n'importe quoi donc effectivement, faut être assez vigilant là où vous prenez les infos » FGD142
	Lack of confidence in sharing personal details (n=6)	« Après, sur les forums, si c'est sur Internet, moi, j'aurais pas une super confiance, de me livrer comme ça (…) que comme tout reste sur Internet, j'aurais plus de mal. » FGD557
	Already access to social and medical network (n=6)	« J'ai des gens autour de moi, à tous niveaux. Si je dois discuter, je peux discuter avec des amis, ma famille, ou autre, sur tel ou tel thème, et au moins, je sais à qui je m'adresse » FG664<br>« Ça remplacera jamais un vrai médecin en face de nous qui saura tous nos antécédents (…) » FGD102
	Negative emotions (n=5)	« Moi, j'ai plutôt arrêté (…) de chercher des informations sur Internet (…) c'était plus une source bah déjà d'erreurs et d'angoisses » FGD313
	Generational gap (n=5)	« Je suis pas sûre que (les plus de cinquante ans, soixante) soient aussi dans la démarche d'aller sur un site internet pour aller voir ce genre d'info (…) je suis pas sûre qu'elle aient accès à tous ces systèmes de communication » FGD438<br>« C'est que les jeunes générations qui vont arriver (a accéder a ce site)… » FGD664
	Not knowing who are you talking to (n=5)	« Ah, bah c'est une catastrophe hein. Vous savez pas qui vous parle. C'est la pire chose. » FG102<br>« Sur les forums (…) (on sait pas) qui se trouve derrière, l'interlocuteur en fait » FGD313
	It's preferable to have face to face exchanges (n=4)	Je sais pas, de voir les gens vraiment en vis-à-vis, enfin en face à face, c'est plus sympa que de faire ça sur internet » FG367<br>« Internet, c'est quand même dénué de rapports humaines, même des forums » FGD307
**Facilitators / Positive aspects or advantages**	Finding useful information (n=6)	« Après, moi, je pense que quand c'est la première mammographie où on est tous hyper inquiètes de faire une mammographie, peut-être d'avoir un truc qui nous explique comment ça va se passer, et ce que c'est, ça peut être pas mal » FGD102
	Trustable sites (n=6)	« Alors, que peut-être effectivement, des associations, ou des centres de recherche mettent sur leur site des noms de forums qui soient légitimés par une instance derrière (…) ça aiderait pas mal pour aller faire ses recherches après. » FGD116

	A safe place (n=6)	« Un groupe fermé qui soit constitué et qui soit modéré par quelqu'un, que ça fasse partie d'une association (…) » FGD139<br><br>« Effectivement, si c'est possible de contrôler (…) Il faut trouver une solution pour être sûr que les femmes se sentent bien (…) la parole des femmes sera vraiment libérée si elles se sentent en confiance. » FGD701
	Having access to a health professional (n=5)	« (…) avoir assez facilemen' l'avi' d'un professionnel, ça peut aider vraiment » FGD217
	Sharing with peers (n=5)	« Parfois ça peut être intéressant par rapport à l'expérience de certaines personnes, leur vécu, je trouve que ça, ça peut aussi aider, de savoir ce qu'elles ont fait dans telles démarches » FGD556

† number of focus groups out of the total number (N=7) in which the theme was mentioned.

3. **Additional information i.e., characteristics and quotations <u>in French</u>, related to women from low socioeconomic background.**

3.1. Participants' characteristics

Supplementary Table 7

	Number of women	%
**Total number**	15	
**Average age** 52		
**Place of residency**		
Dans un village	0	0
Dans une ville	15	100
Dans une grande ville	0	0
Sans réponse	0	0
**Familial status**		
Mariée/ en couple/ pascsée	12	80
Célibataire	1	6.7
Divorcée/séparée	0	0
Veuve	2	13.3
**With children**		
Oui	11	76.5
Non	4	23.5
**Education/School diploma**		
Aucun	4	26.6
BEP/CAP	0	0
BAC	0	0
BAC+2/DUT/BTS/BTSA	0	0
BAC+3	0	0
BAC+4	0	0
BAC+5	0	0
Brevet	1	6.7
Autre	1	6.7
Sans réponse (ne souhaite pas répondre)	9	60

**Occupation**		
Cadre ou profession intellectuelle supérieure	0	0
Profession intermédiaire	0	0
Profession libérale	0	0
Employée	1	6.7
Retraitée	4	26.7
Sans emploi	10	66.6
**With a general practitioner**		
Oui	14	93.3
Sans réponse	1	6.7
**Gynaecological follow-up**		
Oui	10	66.7
Non	5	33.3

3.2. Additional quotations

Supplementary Table 8: breast cancer screening and role of health professionals[††]

Determinants/ Perceptions	Main themes (n)[†]	Quotation
**Perceived susceptibility, severity and benefits**	Screen early to treat early (1 individual) and peace of mind	« Oui. On sait que si la maladie est là, on peut la soigner au début, c'est pas quand ça sera trop tard (…) » FGD1P6<br>« Pour moi (la lettre d'invitation) ça rassure » FGD1P5<br>"La mammographie c'est bien pour contrôler la santé » FGD3M<br>« Ce que je pense, moi, je trouve que c'est très bien de faire une prévention parce qu'on sait jamais ce qui peut arriver » FGD1P7<br>« D'être tranquille. » FGD1P6<br>« c'est bien ..pour contrôler la santé… « FGD3R<br>« L'important c'est contrôler » FGD2B<br><br>« Ça m'angoisse » (elle parle de la lettre) FGD1P6<br>« Je disais, les mots comme ça (cancer du sein), ça fait traumatiser les femmes. » FGD1P6<br>« (cancer sein) Temps en temps ça fait du soucis » GFD2B<br>« J'ai vu cancer dans la lettre (invitation) j'ai eu peur » FGD2N<br>« Mon père et mon frère sont morts de cancer, mon frère poumon, mon frère a la tête…quand j'ai eu un kyste j'ai eu peur » FG3R<br>« Si moi j'ai peur (des cancers)…ça reste toujours caché (note PV : Dans le sens ça prend du temps pour trouver) mon mari est mort de cancer du colon (…) » FG3O<br>« Un jour on peut choper qq chose (cancer)..on fait attention…tu peux pas faire attention pour ça » FGD2R<br>« Quelqu'un en bonne santé…et tout d'un coup ça change » FGD2X<br>« Ça peut arriver) …comme Ma tante et ma belle-sœur …ça va mieux(…).ça peut revenir (le cancer) leur tombe dessus » FGD2Be

	Family history of breast cancer	« Moi, je le fais, systématiquement, c'est tous les deux ans, je crois, parce que j'ai une maman qui a eu un cancer du sein » FG1P7
	Potential symptoms	« …..sentis qq chose j'ai fait des soucis J'ai fait faire mammo » FGD2B<br>« Ça me fait des brûlures…douleur je suis allée voir le médecin » FGD2R
	Recommendations from health professionals*	« Gynécologue m'a dit de faire tout examen faire mammographie faire le colon» FGD3M<br>« J'ai reçu la lettre …je suis allée chez le médecin…c'est elle qui m'a pris un RDV c'était pour la première fois maintenant je vais tout seule » FGD2R<br>« Après on prend l'habitude » FGD2Be<br>« RDV tous seul » FGD2Be<br>« Moi, elle en a jamais parlé. Peut-être qu'elle a beaucoup de demandes parce que souvent ça y a beaucoup de demandes. Elle m'en a jamais parlé. » FGD1P6
Cue to action	Invitation letter	« Ce que je pense, moi, je trouve que c'est très bien de faire une prévention (avec la lettre d'invitation) parce qu'on sait jamais ce qui peut arriver. Et c'est pas la peine d'attendre Octobre Rose pour se faire dépister » FGD1P7<br>« Vient à la maison une lettre comme ça pour contrôler le cancer » FGD30<br>« C'est bien que la lettre vient à la maison, comme ça c'est important…car les personnes travaillent ..pour contrôler..c'est mieux »FGD3R<br>« À la maison » FGD3O<br>« c'est bien quand la lettre vient et ah..on compte pour ça … ..<br>Moi je compte pour ca (recevoir la lettre) (….) je lis pas beaucoup des que je vois la lettre » FGD2R<br>« c'est la date, c'est la date chaque année ….ça vient à tout le monde.recoit ça vient à tout le monde .c'est un rappel » FGD2R<br>« Ma maman recevait la lettre et après elle avait la lettre pour le colon. Après on s'habitue » FGD2B<br>« Ces bon aussi (dans le sens ça marche) aussi pour le sein pour le colon frottis » FGD2N<br>« Il faut lire après (elle parle de la lettre a une autre) il y a des questions » FGD2B<br>« Moi j'aimerais que lettre reste comme ça (vient à la maison) » FGD2R<br>« Oui ça laisse le moral tranquille » FGD2B<br>« Dès que je voie la lettre je vais tout de suite » FGD2N
	Group/community activities or TV adverts	« Suite à intervention CRCDC AuRA à ATELEC avec Christelle » FGD3A<br>« On a eu réunion…dans la salle ici… …voilà il faut faire ca ca..…et un jour elle m'a montré la lettre que j'ai reçu dans la boîte aux lettres…ça aide » FGD2R<br>« (Pub à la télé) c'est bien pour tout le monde…Il y en a qui oublie Des que tu voies une pub..ah est-ce que j'ai fait ? on laisse pas passer la date » FGD2R<br>« (la première fois au sujet cancer sein) La télé quand ils font de la publicité pour aller, pour voir<br>(autre femme :« Je pense que c'est pendant la période d'Octobre Rose.). Voilà. Et là, après, j'ai reçu le courrier » » FGD1P6<br>« Voilà, comme on a fait le groupe avec les femmes avec les autres qui sont venus pour le cancer, la télé, le courrier qu'on envoie. » FG1DP6<br>« Ben pareil, avec les [montre * FGD1P3 et * FGD1P4 du doigt]» (comment elle en a entendu parle) FGD1P7<br>« On a travaillé avec » (elle parle de la lettre d'invitation) FG1P2
Facilitators	Free of cost	« Ben, déjà, c'est gratuit. Donc c'est vrai que ça encourage à le faire tout ça » FGD1P3<br>« En plus c'est gratuit » FGD2B
Habits and past experiences	Experience with mammography	« M'a pris un RDV c'était pour la première fois maintenant je vais tout seule» FGD2N<br>« Après on prend l'habitude (prendre RDV mammo » FGD2be
Empowerment	Individual: to be responsible for one's	« Il faut aller chercher de l'aider et ne pas rester dans son coin » FGD2N

	own health	« Même le médecin c'est 3-4 jours..il faut pas rester il faut trouver une solution » FGD3O
	Individual: to take the lead	« Je suis allée faire traduire lettre…j'avais peur si j'avais eu rdv de prêt avec le médecin je lui aurai parlé (avec assistante sociale) » FGD2N<br>« Obligé…je connais pas je vais chercher quelqu'un pour m'aider..après que j'ai l'habitude je vais toute seule. » FGD2F<br>« Je suis allée comme ça chez gynécologue pour Controller…elle a trouvé kyste elle m'a fait faire mamo » FGD3R<br>« Si elle est pas capable (de comprendre) il faut demander…maintenant il y a tout il y a l'association ..si vous demandez rien faut qu'il cherche une solution »- FG2R<br>« Maintenant j'ai un gynéco, mais avant, j'avais que mon médecin généraliste, c'est lui qui, enfin, c'est moi qui prenais rendez-vous pour justement un contrôle. Mais vraiment que pour ça en fait, pas forcément pour une autre maladie, mais je venais en lui disant bah j'ai. » FG1P4
	Group/ community activities	« Atelec..c'est la première fois que j'ai entendu parler du parler caner »FGD3O<br>« Oui moi jetais avec la dame (atelec) » FGD3R<br>« À Atelec oui…à la maison non » FGD3M<br>« Amis famille…non non» FGD3O<br>« Oui c'était à Atelec »FGD3R<br>« [que les enfants ici la famille est en Belgique FGD3M<br>Je parle pas cancer avec les enfants » FG3M<br>« Toute la famille est au Maroc »FGD3O<br>« Moi ma sœur est en Allemegne»FGD3O<br>« Il y a pas beaucoup d'occasions de parler en français » FGD3R<br>« Tout tout arabe arabe »FGD3O<br>« Moi avant Atelec zéro (français) » FGD2R<br>« La premiere fois que j'ai vu la lettre c'était au cours d'une réunion …ils ont tous expliqué » FGD2R<br>« (la réunion) ça a fait le courage comme ça » FGD2B<br>« Ça aide A » FGD2B, FGD2R<br>« J'ai pas d'Internet, j'ai pas d'ordi, j'ai rien. Non, plutôt, on fait plutôt, excusez-moi, je prends la parole à chaque fois mais, on fait plutôt entre nous [montre les autres participantes du doigt], quand y a des réunions, on en parle, on pose des questions, et puis quand on a des petit-déjeuner santé aussi. Donc c'est plus long, et puis chacun peut poser ses questions, et puis on a un intervenant, une intervenante qui nous explique comment ça se passe le cancer, comment on peut, pas le soigner, mais comment on fait une mammographie tout ça, donc. » FGD1P7<br>« Vous parler avec vos amies la famille les enfants ? (modérateur) vous parler avec vos amies/la famille du cancer du dépistage (modérateur)? collectif FGD3 non non non « ici que les enfants » FGD3M<br>« Moi tous au Maroc »FGD3O<br>« Mais, je trouve que c'est bien qu'il y ait des lettres et qu'il y ait de l'information et qu'on en parle autour de soi. C'est pas toujours tout le monde qui écoute, mais bon, on le dit quand-même. Voilà. » FGD1P7<br>(Est-ce que vous aussi vous avez entendu parler du dépistage autrement que via le médecin ?)<br>« Ben pareil, avec les [montre * FGD1P3 et * FGD1P4 du doigt]. » FGD1P7<br>« Moi, elle en a jamais parlé. Peut-être qu'elle a beaucoup de demandes parce que souvent ça y a beaucoup de demandes. Elle m'en a jamais parlé. Qu'est-ce que vous avez, voilà, voilà. Et la [inaudible] c'est moi que je fais le médicament, je signe [rire]. Voilà, non. Heureusement, y a des autres méthodes pour savoir plus. » FGD1P<br>« Voilà, comme on a fait le groupe avec les femmes avec les autres qui sont venus pour le cancer, la télé, le courrier qu'on envoie. Voilà, on peut faire ça, on peut faire ça. Même si on oublie [inaudible]. » FGD1P6
Barriers	Issues with French language	« Je suis allée faire traduire lettre… (avec assistante sociale) » FGD2N<br>« (accès le problème c'est on comprend pas bien. on parle pas bien on écrit pas bien »FGD3R<br>« Je lis pas beaucoup dès que je vois la lettre.. » FGD2N

	Related to health professionals†	« Ils ont pas le temps avec nous. Il y a toujours du monde, il y a toujours les gens. » FGD1P6<br>« J'ai du suivi, alors y a pas de raison de lui en parler » FGD1P5<br>« [parlent en même temps] Y a toujours du monde. Lorsqu'elle arrive à faire l'ordonnance, c'est déjà beaucoup » FGD1P6<br>« Et parfois ils disent ils n'ont pas le temps les médecins, comme elle explique [pointe à * FGD1P6]. Lorsqu'on arrive [parlent en même temps» FGD1P1<br>« Non, quand tu vas chez le médecin, la première question, pourquoi vous êtes venue ici ? Il t'écris là, il te parle comme ça, [inaudible] au suivant.» FGD1P6
	Pain related to mammography reported but not as a barrier	« Tu as l'impression qu'on t'écrase (…) mais moi ça fait mal » FG1P3<br>« Ça fait mal » FGD2Be<br>« Même pas trois secondes ..après ça va. » FGD2R<br>« Ca fait un peu mal la première fois après ça passe »FGD2B<br>« MA première mammo,,ca fait mal FG2Be<br>« Faire radio comme ça (elle montre) ouh la la » FGD3O<br>« Ça fait mal… ? »non non FGD3 divers<br>« oui « FGD2M<br>« Un petit peu (elle mime la mammo) écrasé » FGD3O<br>(au sujet de la douleur) « Les deux dernières fois, non. (…) Mais autrement, oui, j'avais le temps de le sentir quoi» FG1DP5<br>« [inaudible] ils sont sympas. Heureusement, sinon on va plus y retourner. » FGD1P6 ?<br>« Ouais. Non, mais avant, j'ai assez souffert. » FGD1P5<br>« Ça fait tellement mal, que voilà quoi. Faut vraiment avoir connaissance de tout ça pour se forcer à y aller quoi. » FGD1P3<br>« Il faut y aller et puis c'est pas évident quoi. Il faut prendre un rendez-vous plus y aller, sachant que ça fait mal aussi. » FGD1P3
	Fear of cancer	« Les gens qui veulent pas, c'est la peur qui les, c'est la peur »<br>« La peur d'avoir le cancer, parce que le mot cancer ça fait vibrer les gens » FGD1P6<br>« C'est la peur » FGFD2Be<br>« Vrai, c'est la peur. Du résultat. Je crois que c'est ça, oui » FG1P7<br>« La peur d'avoir le cancer, parce que le mot cancer ça fait vibrer les gens » FGD1P6<br>« Peut-être elle a peur de qq chose » FGD1P3<br>"Quand la personne qui veut pas faire des choses il faut chercher ca sort» FGD2N
Health habits	Mammography	« J'ai reçu la lettre …je suis allée chez le médecin…c'est elle qui m'a pris un RDV c'était pour la première fois maintenant je vais tout seule » FGD2R<br>« Après on prend l'habitude» FGD2Be<br>« RDV tous seul » FGD2Be<br>« Mais comme [plusieurs personnes parlent en même temps]. Maintenant, c'est nous qu'on va automatiquement. Quand on reçoit un courrier, on va. Maintenant, ça va, c'est automatiquement. » FG1P6
Perceptions and expectations of health professionals††	Limit of time in health professionals' † consultations	« (…) les médecins traitants, ils ont pas le temps de dire allez faire le test » FG1P6
	Explain, understand without forcing	« C'est le travail du professionnel de santé de dire que c'est important de faire la mammographie ? » (modérateur) oui collectif FGD3<br>…« Un petit peu» FGD3M<br>« C'est le médecin qui sait ce qu'il faut faire on peut pas rester dans le noir …il va pas montrer tout mais un bout de chemin FGD2R(oui collectif) »<br>« C'est toujours un coup de pouce» FGD2R<br>« Quand on oublie il faut qu'il rappelle FG2R, collectif<br>Faut pas la forcer …mais courage » « c'est le courage » ..peut-être elle a peur de quelque chose..il faut qu'il explique « pourquoi se qui passe » FGD2R<br>« Si la dame veut pas on peut pas la forcer » FGD2Be<br>« Même si elle veut vraiment pas, bah c'est, il insistera pas, mais c'est son rôle de lui expliquer (…) c'est son rôle d'essayer de comprendre » FG1P7<br>« Voilà, être assuré. » * FG1P6

		« Oui. Enfin, d'essayer de la convaincre. De convaincre que c'est pour son bien ».<br>« De convaincre que c'est pour son bien». FGD1P7<br>« De convaincre, mais pas obliger, ça c'est son rôle ». * FGD1P1

† number of focus groups out of the total number (N=3) in which the theme was mentioned.
†† General practitioners and gynecologists mainly

Supplementary Table 9: perceptions of the online tool

Barriers / negative aspects of the proposed online tool	Spreading of misinformation (n=1) †	« Je trouve que Internet des fois, ils exagèrent, ils disent des choses qui font peur (…) Je trouve que des fois, y a des mots, ou des choses qui font peur parce qu'ils exagèrent un peut peut-être aussi. Tu peux pas trop se fier » FG1LSEP7<br>« Parce que des fois qu'on connaît pas les sites, y a certains sites qu'on a dit, c'est pas très rassurant (…) » FG1P1
	Use of connected devices and social network (n=2)	« Moi oui, (j'utilise Internet des fois pour les recherches sur le cancer du sein) parce que quand j'ai un problème je viens voir c'est quoi ça ou pourquoi j'ai ça, est-ce qu'il y a des symptômes (…) tu cherches dans l'Internet, vraiment c'est super » FG1P6
		« Moi j'ai pas d'ordinateur, je regarde sur Internet, sur mon portable, de choses sur la santé, pour les maladies (…) sur Facebook comme moi j'ai un groupe avec les femmes (…) quand quelqu'un qui connaît quelque chose on le voit pour que tout le monde regarde, l'expérience avec des docteurs… » FGD2R
	It's preferable to have face to face exchanges (n=2)	« Moi, non plus, je vais pas sur les sites, moi, si j'ai une question à poser médicale, je préfère parler avec le médecin compétent » FG1P7<br>« Moi non, je vais directement à Ambérieux » FGD22<br>« pour moi c'est bien ; le problème c'est nous pas parler pas comprendre pas écrire le français » FGD3R

† number of focus groups out of the total number (N=3) in which the theme was mentioned.
